# Genetic Analysis of Primaquine Tolerance in a Patient with Relapsing Vivax Malaria

**DOI:** 10.3201/eid1905.121852

**Published:** 2013-05

**Authors:** A. Taylor Bright, Thamer Alenazi, Sandra Shokoples, Joel Tarning, Giacomo M. Paganotti, Nicholas J. White, Stanley Houston, Elizabeth A. Winzeler, Stephanie K. Yanow

**Affiliations:** University of California, San Diego, La Jolla, CA, USA (A.T. Bright, E.A. Winzeler);; University of Alberta Hospital, Edmonton, Canada (T. Alenazi, S. Houston);; King Saud bin Abdulaziz University for Health Sciences, Riyadh, Saudi Arabia (T. Alenazi, S. Houston);; Provincial Laboratory for Public Health, Edmonton, Canada (S. Shokoples, S.K. Yanow);; Mahidol University, Bangkok, Thailand;; University of Oxford, Oxford, UK (J. Tarning, N.J. White);; “La Sapienza” University, Rome, Italy (G.M. Paganotti);; University of Alberta, Edmonton, Canada (S.K. Yanow)

**Keywords:** malaria, Plasmodium vivax, relapse, primaquine, genetics, malaria, parasites

## Abstract

Patients with *Plasmodium vivax* malaria are treated with primaquine to prevent relapse infections. We report primaquine failure in a patient with 3 relapses without any possibility of re-infection. Using whole genome sequencing of the relapsing parasite isolates, we identified single nucleotide variants as candidate molecular markers of resistance.

Of the 5 species of *Plasmodium* that cause human malaria, *P. vivax* has the broadest geographic distribution with 2.85 billion persons at risk throughout the world (*1*). Scientists are becoming increasingly aware of the potential severity of *P. vivax* infections and their effects on public health (*2*). A major challenge is the treatment of the dormant stages, hypnozoites, in the liver. Activation of hypnozoites from this reservoir causes subsequent blood-stage infections, or relapses, weeks to years after the primary infection.

Primaquine (PQ) remains the only approved agent to eliminate hypnozoites. Treatment failure, defined by the occurrence of relapses despite PQ therapy, is often ascribed to inadequate dosing, poor adherence, or reinfection (*3*). However, several cases of PQ tolerance without these confounding factors are reported (*4*,*5*). The mechanism underlying PQ tolerance is not understood, although host and parasite genetic factors are implicated. We describe the genetic analysis of parasite and host markers in a patient with 3 *P. vivax* malaria relapses in a malaria-nonendemic setting where reinfection was not possible.

## The Case

The patient is a 38-year-old man from northeast Africa. In December 2008, he experienced a febrile illness in Sudan that was diagnosed as vivax malaria. He was treated with chloroquine (CQ) but did not receive PQ. The patient recovered and moved to Canada in mid-January 2009. One month after his primary infection, he sought treatment at a hospital in Canada with fever, chills, and malaise. *P.vivax* malaria was diagnosed by microscopy and real-time polymerase chain reaction. He was treated with CQ (600 mg base immediately, 300 mg base at 6, 24, and 48 h), followed by 14 d of PQ (30 mg by mouth daily). His estimated weight was 60 kg. The patient’s symptoms resolved, and smears were negative for *Plasmodium* on day 16. The patient experienced a second episode of symptomatic *P. vivax* malaria 3 months later. He was treated with CQ as before, followed by 28 days of PQ (30 mg by mouth daily). Smears were negative 2 days later. Nearly 30 months later, the patient had a third episode of *P. vivax* malaria. He had not traveled outside North America since his arrival in Canada. He was treated with CQ for 3 d, then PQ for 14 d (30 mg by mouth daily). Smears on days 2 and 9 after CQ treatment were negative. The importance of adherence was emphasized at each clinic visit, and the patient affirms that he took the full course of PQ treatment at the same time every day.

 To identify mutations in parasite genes that are potentially associated with primaquine tolerance, we performed whole genome sequencing on *P. vivax* DNA obtained from patient samples at each relapse (EAC01–03). In total, 55,517 high-confidence single nucleotide variants (SNVs) were genotyped (Technical Appendix). The 3 parasite isolates were genetically related, but not identical, and they have been proposed to be meiotic siblings (A.T. Bright et al., unpub. data).

 In addition, the 3 strains contained SNVs in genes homologous to known *P. falciparum* drug-resistance genes, including *pvdhps*, *pvmdr*, and *pvmrp* (*6*–*8*). Variants compared to the *P. vivax* reference strain SalI, presumed to be primaquine sensitive, were found at 27 of 39 sites within 5 known and putative drug resistance genes (Table). All 3 isolates possessed a double mutant antifolate-resistant genotype in *pvdhfr* (*6*). The SNVs within the putative drug-resistance genes in each of the patient’s 3 samples were identical except at amino acid positions 976 and 1393 of the *pvmdr1* gene. The parasite genomes were also compared to the BrazilI strain of *P. vivax,* which was obtained from a patient who had multiple malaria episodes in a malaria-nonendemic country despite primaquine treatment (*9*). Comparison of the genotypes at the 5 genes demonstrated similar profiles. All strains exhibit intermediate to high levels of antifolate resistance on the basis of mutant genotypes identified in *pvdhfr* and *pvdhps*. In addition, the parasite strains obtained in this study share variant alleles with BrazilI in 2 multidrug resistance–associated transporters, *pvmdr* and *pvmrp*.

**Table:** Genetic polymorphisms in drug-resistance genes from relapsing isolates of *Plasmodium vivax* *

Gene	Chromosome	Polymorphism	Amino acid	Reference	Brazil	EAC01	EAC02	EAC03
pvcrt	1	T330262C	5′ UTR	T	C	C	ND	C
PVX_087980		T330482C	5′ UTR	T	T	T	T	T
		G330484T	5′ UTR	G	G	G	G	G
		C330495A	5′ UTR	C	C	C	C	C
		T331151C	Intron	T	C	C	C	C
		T332453C	Intron	T	C	C	ND	C
		A332874C	Intron	A	C	C	ND	C
		G333391A	Intron	G	G	A	A	A
		A333518G	Intron	A	A	G	G	G
		T333544C	Intron	T	T	C	C	C
pvmrp	2	G153936A	F1629	G	G	ND	A	A
PVX_097025		C154067T	H1586Y	C	C	C	C	C
		G154391A	V1478I	G	A	ND	A	A
		G154567C	G1419A	G	G	G	G	G
		T154646G	Y1393D	T	G	ND	G	G
		T154979A	L1282I	T	T	T	T	T
		T155127G	I1232	T	T	ND	G	G
pvdhfr	5	C964763G	S58R	C	A	G	G	G
PVX_089950		T964796C	Y69	T	C	T	T	T
		G964939A	S117N	G	A	A	A	A
		G964970A	P127	G	G	A	A	A
		A965106C	I173L	A	C	A	A	A
pvmdr1	10	C361917G	K1393N	C	C	C	C	G
PVX_080100		T362031C	K1355	T	T	T	T	T
		A362870G	F1076L	A	A	G	G	G
		G363032A	L1022	G	G	G	G	G
		T363169A	Y976F	T	T	A	A	T
		G363223A	T958M	G	A	A	A	A
		T363374G	M908L	T	G	G	G	G
		G363563A	L845F	G	G	G	G	G
		C364004T	G698S	C	C	T	T	T
		T364509C	T529	T	C	C	C	C
		A364557T	S513R	A	A	T	T	T
		C364598T	D500N	C	T	C	C	C
pvdhps	14	G1256840A	Intron	G	G	A	A	A
PVX_123230		T1257042C	P654	T	T	C	C	C
		G1257064A	A647V	G	G	A	A	A
		G1257856C	A383G	G	C	C	C	C
		G1257859C	S382C	G	C	G	G	G
		C1258389T	M205I	C	T	T	T	T
		T1258579C	E142G	T	T	C	C	C

*UTR, untranslated region; ND, not determined

Host pharmacogenetics may also contribute to PQ failure by affecting drug metabolism. Genetic polymorphisms in the *CYP* gene family are associated with poor or intermediate metabolism of many drugs used to treat tropical infections (*10*) and several of these enzymes are specifically implicated in the metabolism of PQ (*11*) and other antimalarial drugs (*12*). We, therefore, determined whether the patient carried alleles that might also explain the failure of treatment. Based on allele frequencies in northeasernt African populations, polymorphisms within 4 of the 60 *CYP* genes were selected for genotyping: CYP1A2*1C, CYP2B6*6, CYP3A4*1B, and CYP2D6*4 (Technical Appendix). The patient was homozygous for the wild-type allele at all 4 loci.

Lastly, we examined whether the patient metabolized PQ to carboxy-primaquine (CPQ), the main PQ metabolite found in plasma. Drug levels were measured with a stereoselective bioanalytical LC-MS/MS method (W. Hanpithakpong et al., unpub. data). A plasma sample was collected on day 12 of treatment of the 3rd relapse, at 12–15 h post-dose. The total PQ and CPQ concentrations were 90 ng/mL and 1,042 ng/mL, respectively. The measured PQ concentration was similar to simulated maximum concentrations at steady-state in healthy male volunteers (96 ng/mL) and patients with vivax malaria (88 ng/mL). Concentration-time profiles for CPQ could not be simulated because of limited published information. These data demonstrate appropriate absorption of PQ and metabolism into CPQ.

## Conclusions

Although this case highlights the challenges in managing patients with *P. vivax* who relapse after high doses of PQ, it also provides a unique opportunity to clarify the mechanisms underlying PQ tolerance. The multiple relapses in this patient result from previously acquired hypnozoites that likely possessed a genetic profile rendering them tolerant to PQ. Genotyping did not identify any mutations within 4 of the *CYP* loci potentially responsible for the antiparasite effect of PQ and plasma measurements demonstrated adequate levels of PQ and CPQ. However, this study presents a limited screen of polymorphisms in the *CYP2D6* gene (*13*), and we cannot exclude the possibility that other alleles contribute to PQ tolerance.

Parasite genotype data demonstrate that the 3 isolates contain mutations in several putative drug- resistance genes. All 3 isolates are resistant to antifolates and harbor mutations in the ABC transporter genes that are implicated in resistance to numerous antimalarial drugs. Of particular interest are the mutations in the *pvmrp1* gene that encodes a putative multidrug resistance-associated protein. Studies from *P. falciparum* implicate PfMRP1 in glutathione efflux, consistent with the predicted mode of action of PQ in disrupting mitochondrial function (*14*). Furthermore, gene knockouts of *pfmrp1* have increased sensitivity to several antimalarial drugs, including PQ, which suggests this protein may play a role in transporting antimalarial agents out of the parasite (*15*).

This case study demonstrates the feasibility of using molecular tools to better understand therapeutic responses to PQ. Genetic analysis of SNVs in putative resistance genes may identify molecular markers of parasite resistance or correlate with known variations in PQ sensitivity of strains from different geographic areas. Clarification of the role of genetic factors involved in PQ efficacy cannot be readily addressed in populations in which endemic transmission occurs because relapses cannot be distinguished from reinfections. Genetic studies of relapses that occur in nontransmission settings provide a unique opportunity to answer questions about this human pathogen.

Technical Appendix  Supplemental methods and materials are provided, including information on genotyping of cytochrome P450 (CYP) alleles and *Plasmodium vivax*  samples.
