# Supplementary material for: Genetic Analysis of Primaquine Tolerance in a Patient with Relapsing Vivax Malaria
Source: Emerg Infect Dis. 2013 May;19(5):802–5. doi: 10.3201/eid1905.121852 (PMC3647516; doi:10.3201/eid1905.121852)
Supplement: Technical Appendix — Supplemental methods and materials are provided, including information on genotyping of cytochrome P450 (CYP) alleles and Plasmodium vivax samples. [file 12-1852-Techapp-s1.pdf]

Article DOI: http://dx.doi.org/10.3201/eid1905.121852

Genetic Analysis of Primaquine Tolerance in a Patient with Relapsing Vivax Malaria

Technical Appendix

Supplemental Materials and Methods

Ethics Statement

This study was approved by the Health Research Ethics Board of the University of Alberta. The patient provided written informed consent. The consent form states in English that blood samples collected from the patient may be used to genetically characterize the parasites and *CYP* genes from the patient, and that samples may be shared with other researchers for the purpose of investigating the basis of primaquine (PQ) resistance.

Sample Collection

Whole blood samples from the first (EAC01) and second (EAC02) malaria infections were collected in EDTA tubes and stored at –20°C. For the third infection (EAC03), the red blood cell pellet was stored at –80°C. Plasma was collected on day 12 of PQ treatment.

Genotyping of Cytochrome P450 (CYP) Alleles

Human DNA extraction was performed by using the PSS GC12 instrument (Precision System Science Co. Ltd) and eluted into a 100-µL volume. Four alleles were selected for genotyping analysis based on analysis of the literature taking into account the country of origin of the patient and alleles hypothesized to play a role in drug metabolism: *CYP1A2*1C* (Dandara, Basvi, Bapiro, Sayi, & Hasler, 2004), *CYP2B6*6* (Penzak et al., 2007; Wang & Tompkins, 2008), *CYP3A4*1B* (Ferreira et al., 2008; Garsa, McLeod, & Marsh, 2005; Kedmi, Maayan, Cohen, Hauzi, & Rund, 2007) and *CYP2D6*4* (Xie, Kim, Wood, & Stein, 2001).

Regions within the *CYP1A2* and *CYP3A4* genes were amplified by PCR followed by DNA sequencing according to published methods (Nakajima et al., 1999; Paganotti et al., 2011). The *CYP2B6* allele was characterized by PCR-RFLP using the enzyme *BsrI* (Ebeshi, Bolaji, & Masimirembwa, 2011). For *CYP2D6*4*, PCR was performed with the primers: 5′-

CAAGAAGTCGCTGGAGCAGT-3′ (forward) and 5′-GAGGGTCGTCGTACTCGAAG-3′ (reverse) and the following PCR conditions: 94°C for 3 min, 30 cycles of 94°C for 30s, 60°C for 30s, 72°C for 30s, and a final extension step at 72°C for 10 min. PCR products were digested with *Eco*RII and *MvaI* analyzed by RFLP. These enzymes will digest the PCR product when there is a guanine at position 1934 but not when there is an adenine, which corresponds to the *CYP2D6*4* allele. The presence of the mutant allele was further confirmed by direct sequencing of the PCR product.

Parasite Genotyping

For the three parasite samples (EAC01-EAC03), bulk genomic DNA was isolated from frozen whole blood samples using the DNeasy Blood and Tissue kit (Qiagen) as per the manufacturer's instructions. Whole genome capture of parasite DNA for the three samples was performed as described previously (Bright et al., 2012). Captured DNA was paired-end sequenced on an Illumina HiSeq 2000 for 101 bp per read plus one 7-bp index read using Illumina v.3 chemistry. Data for each sample sequenced in this study is available in the NCBI Sequence Read Archive [SRA057904]. Fastq files obtained from sequencing were aligned to the Sal1 reference using BWA (v. 0.5.9) (Li & Durbin, 2009). Aligned reads were cleaned and analyzed by using Picard (v. 1.51) and GATK (v. 1.6+) (DePristo et al., 2011). 55,517 high confidence SNVs were genotyped in all three samples using GATK (Bright et al., submitted). Heterozygous SNV calls were excluded from downstream analysis.

Measurement of Drug Levels

Plasma concentrations of PQ and its major metabolite, carboxy-primaquine (CPQ), were measured with a newly developed stereoselective bioanalytical method (Hanpithakpong et al., manuscript in preparation). In summary, the method used solid-phase extraction followed by liquid chromatography coupled to tandem mass spectrometry. Triplicates of 3 quality control samples were analyzed in the same batch to ensure that accuracy and precision were acceptable according to United States Food and Drug Administration (FDA) standards (FDA Guidance for Industry–Bioanalytical Method Validation). Measured drug concentrations were compared to simulated concentration-time profiles based on literature values for pharmacokinetic parameters in healthy male volunteers (Binh et al., 2009; Cuong et al., 2006; Elmes, Bennett, Abdalla, Carthew, & Edstein, 2006; Fletcher et al., 1981; Mihaly et al., 1985; Mihaly, Ward, Edwards,

Orme, & Breckenridge, 1984) and male patients with vivax malaria (Bangchang, Songsaeng, Thanavibul, Choroenlarp, & Karbwang, 1994.; Bhatia et al., 1986; Kim et al., 2004).

References

Bangchang KN, Songsaeng W, Thanavibul A, Choroenlarp P, Karbwang J. Pharmacokinetics of primaquine in G6PD deficient and G6PD normal patients with vivax malaria. Trans R Soc Med Hyg. 1994;88:220–2.. http://www.ncbi.nlm.nih.gov/pubmed/8036681

Bhatia SC, Saraph YS, Revankar SN, Doshi KJ, Bharucha ED, Desai ND, et al. Pharmacokinetics of primaquine in patients with *P. vivax* malaria. Eur J Clin Pharmacol. 1986;31:205–10. PubMed http://dx.doi.org/10.1007/BF00606660

Binh VQ, Chinh NT, Thanh NX, Cuong BT, Quang NN, Dai B, et al. Sex affects the steady-state pharmacokinetics of primaquine but not doxycycline in healthy subjects. Am J Trop Med Hyg. 2009;81:747–53.. PubMed http://dx.doi.org/10.4269/ajtmh.2009.09-0214

Bright AT, Tewhey R, Abeles S, Chuquiyauri R, Llanos-Cuentas A, Ferreira MU, et al. Whole genome sequencing analysis of *Plasmodium vivax* using whole genome capture. BMC Genomics. 2012;13:262. PubMed http://dx.doi.org/10.1186/1471-2164-13-262

Cuong BT, Binh VQ, Dai B, Duy DN, Lovell CM, Rieckmann KH, et al. Does gender, food or grapefruit juice alter the pharmacokinetics of primaquine in healthy subjects? Br J Clin Pharmacol. 2006;61:682–9. PubMed http://dx.doi.org/10.1111/j.1365-2125.2006.02601.x

Dandara C, Basvi PT, Bapiro TE, Sayi J, Hasler JA. Frequency of −163 C>A and 63 C>G single nucleotide polymorphism of cytochrome P450 1A2 in two African populations. Clin Chem Lab Med. 2004;42:939–41. PubMed http://dx.doi.org/10.1515/CCLM.2004.152

DePristo MA, Banks E, Poplin R, Garimella KV, Maguire JR, Hartl C, et al. A framework for variation discovery and genotyping using next-generation DNA sequencing data. Nat Genet. 2011;43:491–8. PubMed http://dx.doi.org/10.1038/ng.806

Ebeshi BU, Bolaji OO, Masimirembwa CM. Allele and genotype frequencies of cytochrome P450 2B6 and 2C19 genetic polymorphisms in the Nigerian populations: possible implications on anti-retroviral and anti-malarial therapy. International Journal of Medicine and Medical Sciences. 2011;3:193–200.

Elmes NJ, Bennett SM, Abdalla H, Carthew TL, Edstein MD. Lack of sex effect on the pharmacokinetics of primaquine. Am J Trop Med Hyg. 2006;74:951–2. http://www.ncbi.nlm.nih.gov/pubmed/16760502 PubMed

Ferreira PE, Veiga MI, Cavaco I, Martins JP, Andersson B, Mushin S, et al. Polymorphism of antimalaria drug metabolizing, nuclear receptor, and drug transport genes among malaria patients in Zanzibar, East Africa. Ther Drug Monit. 2008;30:10–5. PubMed http://dx.doi.org/10.1097/FTD.0b013e31815e93c6

Fletcher KA, Evans DA, Gilles HM, Greaves J, Bunnag D, Harinasuta T. Studies on the pharmacokinetics of primaquine. Bull World Health Organ. 1981;59:407–12. http://www.pubmedcentral.nih.gov/articlerender.fcgi?artid=2396059&tool=pmcentrez&rendertype=abstract. PubMed

Garsa AA, McLeod HL, Marsh S. CYP3A4 and CYP3A5 genotyping by pyrosequencing. BMC Med Genet. 2005;6:19. PubMed http://dx.doi.org/10.1186/1471-2350-6-19

Kedmi M, Maayan S, Cohen SB, Hauzi M, Rund D. MDR1 and CYP3A4 polymorphisms are associated with HIV seropositivity in Israeli patients but do not influence the course of HIV disease. AIDS Patient Care STDS. 2007;21:653–8. PubMed http://dx.doi.org/10.1089/apc.2006.0148

Kim Y-R, Kuh H-J, Kim M-Y, Kim Y-S, Chung W-C, Kim S-I, et al. Pharmacokinetics of primaquine and carboxyprimaquine in Korean patients with vivax malaria. Arch Pharm Res. 2004;27:576–80. http://www.ncbi.nlm.nih.gov/pubmed/15202566 PubMed http://dx.doi.org/10.1007/BF02980134

Li H, Durbin R. Fast and accurate short read alignment with Burrows-Wheeler transform. Bioinformatics. 2009;25:1754–60. PubMed http://dx.doi.org/10.1093/bioinformatics/btp324

Mihaly GW, Ward SA, Edwards G, Orme ML, Breckenridge AM. Pharmacokinetics of primaquine in man: identification of the carboxylic acid derivative as a major plasma metabolite. Br J Clin Pharmacol. 1984;17:441–6. http://www.pubmedcentral.nih.gov/articlerender.fcgi?artid=1463409&tool=pmcentrez&rendertype=abstract PubMed http://dx.doi.org/10.1111/j.1365-2125.1984.tb02369.x

Mihaly GW, Ward SA, Edwards G, Nicholl DD, Orme ML, Breckenridge AM. Pharmacokinetics of primaquine in man. I. Studies of the absolute bioavailability and effects of dose size. Br J Clin Pharmacol. 1985;19:745–50. http://www.pubmedcentral.nih.gov/articlerender.fcgi?artid=1463857&tool=pmcentrez&rendertype=abstract. PubMed http://dx.doi.org/10.1111/j.1365-2125.1985.tb02709.x

Nakajima M, Yokoi T, Mizutani M, Kinoshita M, Funayama M, Kamataki T. Genetic polymorphism in the 5′-flanking region of human CYP1A2 gene: effect on the CYP1A2 inducibility in humans. J Biochem. 1999;125:803–8. http://www.ncbi.nlm.nih.gov/pubmed/10101295 PubMed http://dx.doi.org/10.1093/oxfordjournals.jbchem.a022352

Paganotti GM, Gallo BC, Verra F, Sirima BS, Nebié I, Diarra A, et al. Human genetic variation is associated with *Plasmodium falciparum* drug resistance. J Infect Dis. 2011;204:1772–8. PubMed http://dx.doi.org/10.1093/infdis/jir629

Penzak SR, Kabuye G, Mugyenyi P, Mbamanya F, Natarajan V, Alfaro RM, et al. Cytochrome P450 2B6 (CYP2B6) G516T influences nevirapine plasma concentrations in HIV-infected patients in Uganda. HIV Med. 2007;8:86–91. PubMed http://dx.doi.org/10.1111/j.1468-1293.2007.00432.x

Wang H, Tompkins LM. CYP2B6: new insights into a historically overlooked cytochrome P450 isozyme. Curr Drug Metab. 2008;9:598–610. http://www.pubmedcentral.nih.gov/articlerender.fcgi?artid=2605793&tool=pmcentrez&rendertype=abstract PubMed http://dx.doi.org/10.2174/138920008785821710

Xie HG, Kim RB, Wood AJ, Stein CM. Molecular basis of ethnic differences in drug disposition and response. Annu Rev Pharmacol Toxicol. 2001;41:815–50. PubMed http://dx.doi.org/10.1146/annurev.pharmtox.41.1.815
